# Quality-Shaping Factors and Endodontic Treatment amongst General Dental Practitioners with a Focus on Denmark

**DOI:** 10.1155/2012/526137

**Published:** 2012-03-26

**Authors:** Sune Demant, Merete Markvart, Lars Bjørndal

**Affiliations:** Section of Cariology and Endodontics, Institute of Odontology, University of Copenhagen, Nørre Allé 20, 2200 Copenhagen N, Denmark

## Abstract

There is a gap between the endodontic outcome that can be achieved and the outcome observed on the basis of worldwide general dental practitioner data. The quality of root canal treatment (RCT) is shaped by the dentist's knowledge, attitude, and skills, but it may also be influenced by the patient's demands and degree of satisfaction. The topic has only been sparsely investigated. Although dental health has increased over the years in Denmark, the number of performed root fillings has also increased, probably because the number of tooth extractions have declined and more molar teeth have been treated. Caries appears to be the main cause for performing RCT and a preventive approach by employing stepwise excavation may reduce RCT, but this strategy does not remove the gap. Factors influencing RCT quality could be the status on adoption of nickel-titanium rotary technology, more focus on infection control (rubber dam use, knowledge of factors important for prognosis), as dentists often think that they are good at doing RCT, but often perform inadequately, an alteration of clinician's awareness of their performance in the context of dental practices, seems warranted. Finally, the development of new preventive modalities for pulp and apical inflammation are crucial.

## 1. Introduction

It is well known from endodontic textbooks [1] and clinical studies conducted in controlled environments [2–4] that the prognosis for conventional orthograde root canal treatment is good. Performing pulpectomy results in a successful outcome in between 90 and 95% of treated patients. The definition of a successful treatment outcome is sound periapical conditions after 1-year follow-up as reflected by radiography and when the patients do not report any subjective symptoms. The expectation is somewhat lower in cases where the patients have a necrotic root canal and bacterial infection, leading to periapical inflammation with periapical radiolucency, as evidenced on radiographs. The bacteria-induced periapical inflammation can be expected to heal in between 80 and 85% or even more [5], in patients receiving root canal treatment, which means that the apical radiolucency has diminished after follow-up of 1–4 years and the patients do not have subjective symptoms. 

Is it possible to achieve similar outcomes when treatment takes place within a general dental practice environment? Radiographically based epidemiological data covering root canal treatment amongst general dental practitioners indicates that the relatively high outcome rates are seemingly difficult to reach [1]. Many international studies, not only in Scandinavian countries, have shown that there is a close association between the technical quality of a root filling and the prevalence of apical periodontitis. Danish data [6] have shown on the basis of subpopulations that the vast majority of the examined root canal fillings were of suboptimal quality. 59% of the root-filled teeth had insufficient lateral seal and 40% wrong length of the root filling. Moreover, apical radiolucency was present in 52% of the root-filled teeth. Notably, there is a gap between what it is possible to achieve in relation to endodontic treatments and what is carried out in a general practice environment. This paradox has been documented in many populations worldwide [1]. The quality of endodontic treatments is shaped by the dentist's knowledge, attitude, and skills, but it may also be influenced by the patient's demands and degree of satisfaction as well as by the platform within society. For example, the dental service in a given society might be partly funded by national or private health insurance systems, which in reality may determine whether a specific intervention is performed in practice. In general, the quality-shaping factors that influence endodontic treatment in a dental practice environment have only been sparsely investigated. To reduce the gap between the endodontic treatment outcome that can be achieved and the outcome observed on the basis of general dental practitioner data, the following questions appear relevant. What is the status of the etiology of apical periodontitis? What is the frequency of root canal treatments during the past few decades, and what are the reasons today for carrying out root canal treatments in general practice? Would it be possible to prevent any of these reasons? Finally, what is the status of the endodontic routine amongst general dental practitioners in terms of knowledge, attitudes, and skills?

## 2. The Causal Significance of Apical Periodontitis and Bacterial Infection

General dental practitioners should attempt to achieve the best outcome rate within the field of endodontic treatment, because a high level of knowledge is currently available concerning the etiology and pathogenesis of both pulpitis and apical periodontitis. The main cause for the development of disease in the pulp and the apical periodontium is bacterial infection. Other conditions may be listed such as: trauma, iatrogenic injuries, trauma following tooth preparation, as well as potential toxic injuries from dental materials. However, if any of these conditions should cause apical periodontitis to become visible on a radiograph, it would be associated with bacterial infection [1].

 The classical rat study by Kakehashi and coworkers [7] is very instructive for a proper understanding of the etiology of apical periodontitis. The study showed the causal significance of bacterial infection. The effect of pulp exposure was compared between normal rats and rats placed within a bacteria-free environment. All rats with exposed pulps in a normal environment got severe inflammation and necrosis due to bacterial invasion, followed by apical inflammation. In contrast, in the rats placed in an environment without bacteria, all the exposed pulps showed tertiary dentinogenesis with virtually no evidence of pulp inflammation. The causal significance of bacterial infection for the development of periapical inflammation was subsequently demonstrated in primates and humans, and the understanding is today much more detailed [1, 8]. Overall, root canal treatments can be seen as procedures that lead to either treatment or prevention of microbial root canal infection.

## 3. Frequency of Root Canal Treatment during the Past Few Decades

**Figure 1:**
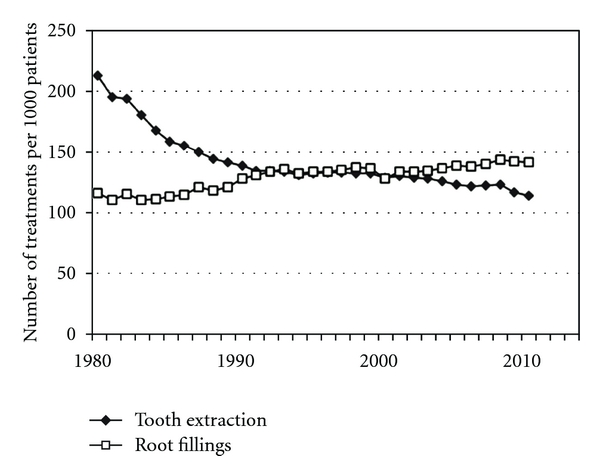
The number of root fillings and tooth extractions per 1000 patients from 1977 to 2010. (Source: Danish Dental Association 2011.)

Dental health has increased during the past few decades [9]. It could be speculated that the number of root canal treatments may have declined correspondingly and that caries may not be the main reason for carrying out endodontic treatment. However, based on more than 30 years of records in Denmark using annual treatment statistics from the Danish Dental Association and the National Health Insurance (Figure 1), it is apparent that a 22% increase has occurred in the number of root fillings per 1000 patients (115 root fillings compared with 140 root fillings). A deeper analysis of the treatment statistics has previously been published for the period 1977–2003 [10]. The increase includes the treatment of multirooted teeth, and the majority of the root canal treatments are carried out in adults aged between 40 and 59 years. On the basis of this Danish nationwide database, it is not possible to confirm a decline in the number of root fillings. This may partly be explained by the fact that a marked reduction in the number of tooth extractions occurred in the same period, bringing many more teeth into the total population of teeth which might potentially undergo endodontic treatment. Finally, more multirooted teeth have been root-filled than previously. A similar trend has been observed epidemiologically by comparing two Danish populations from the 1970s to the 1990s, where molars were the most frequent root-filled tooth group [11]. In Sweden during a 20-year period, it was also possible to show not only an increased number of root fillings, but also more teeth with apical periodontitis [12].

## 4. Reasons for Performing Root Canal Treatments

The most frequent reason for performing root canal treatments within a Danish practice-based environment is caries in a vital tooth (55%), followed by infractions (14%). These data are based on the responses to a questionnaire from 600 randomly selected general dental practitioners [13]. Retreatment was hardly ever carried out, which appears surprising as it is well documented that there is a large pool of suboptimal root fillings within the populations [1]. Based on several studies confirming the same trend, endodontic treatment still covers a large part of the work within dental care [11]. It has become more complex as it is carried out more often in multirooted teeth and caries is still the main reason for performing root canal treatment.

## 5. May Deep Caries among Adults Be Treated by an Endodontic Preventive Treatment Strategy?

Caries appears to be the main reason for performing root canal treatment in vital teeth. Would it be possible to raise the quality of root canal treatments by reducing the number of root canal treatments following caries treatment, thereby decreasing the number of endodontic complications? The potential of an endodontic preventive strategy of treating deep caries among adults was recently investigated in a randomized clinical multicenter trial [14]. The stepwise excavation procedure was compared with one completed excavation, and two pulp-capping procedures were randomly selected and compared in patients (direct pulp-capping versus partial pulpotomy), where excavation had led to pulp exposure.

Stepwise excavation was significantly better for preventing exposure of the pulp than one completed excavation. The number of patients without exposure of the pulp and vital pulp without apical radiolucency after *∼*1-year follow-up was significantly higher in the stepwise excavation group (74.1%) versus the one completed excavation group (62.4%). In patients where the pulp-capping procedures were carried out, both intervention groups had very low pulp survival rates (direct pulp-capping 31.8% versus partial pulpotomy 34.5%) following *∼*1-year follow-up. The majority of these capped treatments failed due to pain within the first year.

The beneficial effect of using stepwise excavation can be expressed by an absolute risk reduction of 11.7% or by “numbers needed to treat.” This means that the clinician will avoid 1 pulp exposure by using the stepwise excavation approach as opposed to the one completed excavation following every 8 or 9 deep caries treatment. Today it appears that this present trial is one of the few high-quality randomized clinical trials amongst adults that deals with the treatment of deep caries, but more high-quality randomized clinical trials are needed [15–17].

Neither of the two pulp-capping procedures within the above-mentioned multicenter trial [14] led to promising results. Both procedures led to a high frequency of failed treatments (*∼*67%). Clinically, these results indicate that each time the clinician caps 2 deep caries lesions involving more than 3/4 of the dentin (as examined on a radiograph) one of the treatments will suffer from pain or another complication such as pulp necrosis or apical periodontitis. In addition, it was not possible to indicate a difference between the two capping procedures because the number of patients was too small.

## 6. The General Dental Practitioner's Knowledge and Attitude to Prognosis in relation to Root Canal Treatment Procedures

It seems unrealistic to imagine that endodontic treatments following deep caries treatment can be completely prevented based on the caries trial referred to above [14]. Therefore, discussion concerning quality-shaping factors in relation to root canal treatments is necessary. An important factor could be the knowledge of general dental practitioners regarding the prognosis of root canal treatment.

### 6.1. Knowledge of Prognostic Conditions

**Figure 2:**
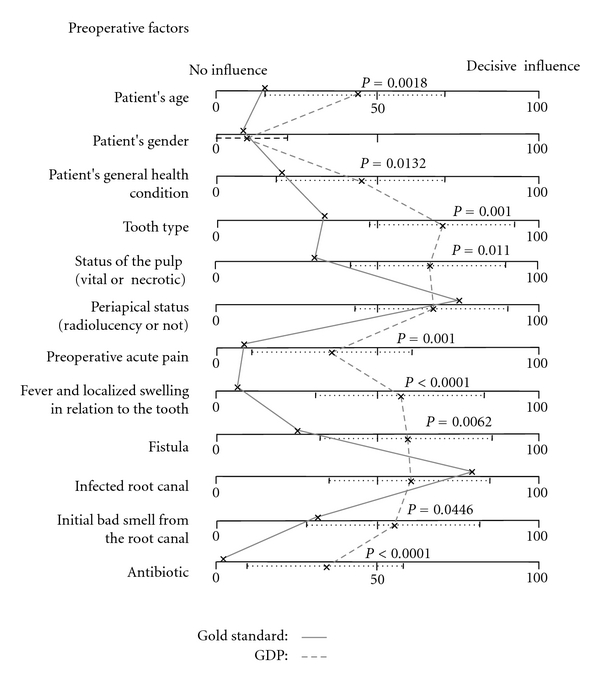
The gold standard on preoperative factors affecting endodontic outcome compared to the GDP (General Dental Practitioner) group response. *P* values denote the significant results from the Wilcoxon tests. (Reprinted with permission from OOOOE, Elsevier Inc., Philadelphia, PA, USA, [18].)

A group of randomly selected general dental practitioners was asked in a questionnaire [18] about the potential influence of preoperative, operative, and postoperative factors on prognosis. The same factors were evaluated by a group of endodontic researchers. These experts were selected on the basis of a literature search presenting the most productive authors within the field of endodontic outcome studies. A gold standard (GS) for each of the factors was constructed on the basis of the expert group response. Both the general dental practitioners and the expert group were asked to judge each prognostic factor based on a Visual Analogue Scale. 0 meant that the factor in question did not have any influence on prognosis, whereas the value 100 represented a decisive influence on the prognosis. The results indicated that many of the preoperative factors were overestimated by the dentists as having an important influence on prognosis. In particular, there was a high focus on “acute clinical symptoms,” whereas the GS emphasized “periapical status” and “bacterially infected root canals” as having a decisive influence on treatment prognosis (Figure 2). The study [18] showed that the performance of suboptimal root canal treatments amongst general dental practitioners may be associated with their insufficient knowledge about factors believed to be important for a good prognosis following root canal treatment.

The data on general dental practitioners confirms the so-called “praxis based theory” [19], because the general dental practitioner is obviously not following a gold standard. The “praxis concept theory” hypothesizes that the general practitioners imagine periapical health and disease, not as either/or situations but as stages on a continuous scale. The cut-off point for the decision to treat is value-dependent, resulting in a huge interindividual variation between practitioners.

The evaluation of preoperative factors having a decisive influence for the outcome plays an important part in the clinical decision making process. An illness-focused strategy [20, 21] seems to attract the majority of Danish general dental practitioners, as many of the preoperative factors believed to impair the endodontic outcome were related toward acute symptoms of infection, that is, as long as the patient does not complain or show any clinical symptoms of periapical disease the treatment result is accepted. A focus on uncomfortable clinical symptoms was also noted among a small group of Swedish practitioners in their decision-making on whether or not to retreat a root-filled tooth [21].

## 7. Nonadoption of New and Old Endodontic Technology

Today more is known concerning the skills and attitudes among Danish general dental practitioners with respect to the routine root canal treatment procedure [22]. For example, obtaining an aseptic working field was regarded by practitioners as being the most difficult procedure to carry out, whereas the root canal treatment *per se* was not assessed as being a particular difficult sequence and it was also assessed as being carried out quite fast. The vast majority of the general dental practitioners assessed themselves as being at an “excellent” or “satisfactory” level of skills with respect to “root canal preparation procedure” and “root filling procedure,” whereas as many as 40% of the involved practitioners regarded their microbiological knowledge as not up to standard. A similar survey was carried out involving endodontic attitudes and skills amongst dental practitioners in Scotland [23]. Most of the dentists reported high confidence in endodontic diagnostics as well as in treating endodontic pathology. However, the actual pattern of the endodontic treatment profile in fact revealed many poorly performed root fillings with the presence of apical periodontitis. A plausible explanation for this false sense of security could be that almost every second dentist never performed radiographic follow-up of their root canal treatments.

### 7.1. The Use or Nonuse of Rubber Dam

 Several studies have shown that only a small part of the general dental practitioner environment uses rubber dam as an integral part of the aseptic working field during endodontic treatment [24], even though international guidelines [25], universities, and national dental association recommendations unanimously stress that it is obligatory. Based on the causal bacterial relationship for the etiology of periapical pathology, it is difficult to understand the pattern noted within the general practitioner environment. Firstly, it appears unwise to avoid rubber dam, as it provides a safeguard against the potential loss of instruments and medicament into the throat. Secondly, studies have shown that the avoidance of rubber dam may lead to the nonuse of sodium hypochlorite as the root canal irrigation agent, and instead other alternative agents are applied without the same documentation on their antibacterial effect [24]. Based on a questionnaire, the attitudes of final-year dental students to the use of rubber dam showed that more than 50% of the students predicted that their use of rubber dam would decrease once they were in independent practice [26]. This underlies the need to maintain the awareness of both dental students and general dental practitioners of the need to use rubber dam [26]. The frequent reasons for justifying the nonuse of rubber dam are not confirmed in the literature [24]. Patients' dislike of the use of rubber dam is not documented either in children or adults. It appears that it is the attitude of the general dental practitioners and not the attitude of the patients that is the decisive element for nonuse of rubber dam.

It may be claimed that better clinical evidence is needed for the use of rubber dam. However, the endodontic community would never initiate a huge and expensive randomized clinical trial comparing the use versus nonuse of rubber dam, as no previous clinical report has ever justified a nonuse approach. A meta-analysis was performed on the basis of observational studies describing the success rate of endodontic treatment of teeth with vital and nonvital pulps [27]. In this analysis, one study [28] had a markedly lower success rate (approx. 20%) than the others. Taking into account the methodological problems of comparing these studies, in that same study, it was reported that a rubber dam procedure was not used in *∼*50% of the treatments. This seems to be one of the few studies documenting the status of an uncontrolled aseptic working field. Several conditions within the Danish system bring hope that the curve of nonusers of rubber dam will change. A lot of attention has been devoted to explaining why it should be used: preparation of an aseptic working field, improved visible contrast, and so forth. A relatively new contract has also been introduced between the Danish Dental Association and the Danish National Health Insurance (where the fixed fee for root canal treatment was abandoned) and this has considerably decreased a potential time-cost dilemma. Thus, Danish general dental practitioners today have a remuneration system that could give an adequate reward for quality, because an individual fee can be introduced reflecting the actual costs of equipment, time, and so forth. Finally, it has been shown that if dentists use several endodontic technologies (apex locators, nickel titanium instruments), they are also more frequent users of rubber dam. It can be described as a “cluster” effect, which may bring about a renaissance in the use of rubber dam [22].

### 7.2. Adoption of Nickel-Titanium Instruments during Root Canal Preparation

Five years ago a low rate of adoption (10%) of nickel-titanium rotary instruments was noted amongst general dental practitioners in Denmark [22], although root canal preparation using stainless steel instrumentation is today considered an outdated standard. Clinical studies show that the use of nickel-titanium rotary protocols produces fewer procedural errors and may also produce an enhanced clinical outcome [29]. The shift from stainless steel instrumentation toward rotary instruments may be improved [30] when practitioners are offered an educational package including hands-on training and lectures dealing with nickel-titanium technology. A long-term effect is reached concerning root-filling quality; however, the technology shift alone will not eliminate clinical work of substandard quality [31].

### 7.3. The Role of the Patient as Viewed from Endodontic Claims

 In the interplay between the dentist and the patient, the content of a patient complaint can be used to describe whether suboptimal root canal treatment may be a visible problem among patients [32–34]. In Danish claim material collected over a 10-year period, the second most frequent malpractice claim category was endodontic treatment. The most frequent reason for suboptimal endodontic treatment was technical shortcomings and technical treatment complications. Male dentists and female patients were overrepresented in the material indicating a sex influence on aspects of the patient-dentist communication that may be important for liability claims. No specific attention was paid to the importance of an aseptic technique during root canal treatment in the available reports from the complaint boards. Thus, the focus on endodontic infection control seems not yet entirely integrated between the complaint board platforms and the universities in Denmark.

## 8. Conclusions

Endodontic treatment is frequently performed, and caries is still the main reason for performing root canal treatment. Potential factors influencing the “gap” between the endodontic healing rates that can be achieved and those found in most populations treated by general practitioners may be:

- a low rate of adoption of new technology among general dental practitioners;
- no systematic evidence of general dental practitioners' awareness of microbial topics that influence the development of apical periodontitis (such as mandatory use of cleansed and disinfected rubber dam and/or lack of awareness of preoperative factors that are important in determining and controlling the outcome of root canal treatment);
- the vast majority of general dental practitioners disclose a high level of confidence in performing endodontic treatments; however, suboptimal root filling quality may be accepted as long as it prevents symptoms;
- endodontic-related claims were the second most frequent category within a large claim material covering 10 years and perceived technical shortcomings dominated the endodontic complaints. Substandard root canal treatments are not invisible clinical procedures for the patient.

## 9. Clinical Implications and Future Prospects

For the prevention of endodontic treatments in adults with deep caries, a stepwise excavation approach versus a direct complete excavation approach should be recommended. Recently investigated pulp-capping procedures had low success rates, and whether these procedures should be performed at all in deep carious exposed adult teeth is questioned. When general dental practitioners perform root canal treatments they seem to know what they should do, think that they are good at doing it, but often perform inadequately as indicated from epidemiological data. Thus, a mandatory application of follow-up procedures after endodontic treatment seems crucial and might alter clinicians' awareness of their performance in the context of dental practices.

In order to reduce the “gap,” ongoing adoption of new advances is central in preventing both pulp and apical inflammation. Finally, implementation of endodontics as a speciality seems warranted in countries where this is still not the case in order to optimize the spread of quality-shaping factors.
